# Influence of TMX2-CTNND1 polymorphism on cortical thickness in schizophrenia patients and unaffected siblings: an exploratory study based on target region sequencing

**DOI:** 10.47626/1516-4446-2023-3322

**Published:** 2024-03-25

**Authors:** Wenjian Tan, Yixin Cheng, Danqing Huang, Dayi Liu, Jiamei Zhang, Jinyue Li, Zhening Liu, Yunzhi Pan

**Affiliations:** Department of Psychiatry, National Clinical Research Center for Mental Disorders and National Center for Mental Disorders, The Second Xiangya Hospital of Central South University, Changsha, Hunan, China

**Keywords:** Schizophrenia, cortical thickness, genetics

## Abstract

**Objective:**

The advancement of neuroimaging and genetic research has revealed the presence of morphological abnormalities and numerous risk genes, along with their associations. We aimed to estimate magnetic resonance imaging-derived cortical thickness across multiple brain regions.

**Methods:**

The cortical thickness of 129 schizophrenia patients, 42 of their unaffected siblings, and 112 healthy controls was measured and the candidate genes were sequenced. Comparisons were made of cortical thickness (including 68 regions of the Desikan-Killiany Atlas) and genetic variants (in 108 risk genes for schizophrenia) among the three groups, and correlation analyses were performed regarding cortical thickness, clinical symptoms, cognitive tests (such as the N-back task and the logical memory test), and genetic variants.

**Results:**

Schizophrenia patients had significantly thinner bilateral frontal, temporal, and parietal gyri than healthy controls and unaffected siblings. Association analyses in target genes showed that four single nucleotide variants (SNVs) were significantly associated with schizophrenia, including thioredoxin-related transmembrane protein 2-catenin, cadherin-associated protein, delta 1 (SNV20673) (positive false discovery rate [P_FDR_] = 0.008) and centromere protein M (rs35542507, rs41277477, rs73165153) (P_FDR_ = 0.030). Additionally, cortical thickness in the right pars triangularis was lower in carriers of the SNV20673 variant than in non-carriers (P_FDR_ = 0.048). Finally, a positive correlation was found between right pars triangularis cortical thickness and logical memory in schizophrenia patients (r = 0.199, p = 0.032).

**Conclusions:**

This study identified regional morphological abnormalities in schizophrenia, including the right homologue of Broca’s area, which was associated with a risk variant that affected delta-1 catenin and logical memory. These findings suggest a potential association between candidate gene loci, cortical thickness, and schizophrenia.

## Introduction

Schizophrenia, a mental disorder that causes significant disability, is up to 80% heritable according to family and twin studies.^1^ However, the prevalence of schizophrenia can hardly be explained by a monogenic inheritance pattern. The pathogenesis of schizophrenia is likely related to multiple genetic variations with small to moderate effects. Over the past decade, genome-wide association studies have been performed in schizophrenia to investigate its genetic mechanism, including ultra-large-scale international studies with a global impact. The Psychiatric Genomics Consortium’s phase 2 genome-wide association study identified 108 independent loci in schizophrenia,^2^ and 287 significant genome-wide loci have been found in its phase 3 study.^3^ This study, which included both East Asian and European populations, found a mostly overlapping genetic basis for schizophrenia between the two populations,^4^ suggesting that it has a complex but stable genetic mechanism. At this point, thousands of schizophrenia- related candidate variants have been repeatedly identified.^5^ In the gene-brain-schizophrenia framework, these studies only suggest an association between genes and diseases. However, the association between each candidate variant and cortical morphology is still unclear. Hence, it is meaningful to study the biological pathways between risk genes, brain abnormalities, and schizophrenia, which could help clarify the whole process from a micro to a macro level.

Schizophrenia is also widely accepted as a brain disorder involving abnormal brain structure. Neuroimaging studies have revealed abnormal cortical morphology in schizophrenia patients.^6^ Large-scale studies have reported robust findings of cortical thinning in the frontal and temporal lobe regions.^7^ Based on the radial unit hypothesis of cortical development,^8^ the number of neurons in the cortical column of the cerebral cortex determines the cortical thickness, and different neurodevelopment programs shape and regulate cortical thickness. This biological process is regulated by genetic variants. Recently, two independent teams, using genetically-informed atlases and traditional non-genetic atlases, found that genetic polymorphisms were closely associated with cortical development, such as cortical thickness.^9^,^10^ Thus, cortical morphology is a promising intermediate between genetic variants and phenotype. A study using a mixed linear model found that polygenic risk scores (PRS) correlated with cortical volume, surface area, and cortical thickness, and that PRS was significantly negatively correlated with insular cortex thickness, although these findings were in healthy people.^11^ Notably, one study investigated shared genetic variations between schizophrenia and cortical thickness, finding that a substantial proportion of single nucleotide variants (SNVs) associated with total surface area and mean cortical thickness were also associated with schizophrenia risk.^12^ In this study, we further explored the association between cortical phenotype and schizophrenia genotype in an identical patient sample.

Unaffected siblings of schizophrenia patients are an ideal population for studying intermediate phenotypes because they are healthy carriers of risk genes. Unaffected siblings of schizophrenia patients have a greater-than-average genetic propensity to schizophrenia, which could contribute to higher levels of psychosis symptoms. Indeed, siblings of schizophrenia patients have been found to have higher rates of all schizophrenia spectrum personality disorders than siblings of healthy volunteers.^13^ Moreover, it has been reported that siblings of schizophrenia patients have worse cognitive performance than healthy controls.^14^ A review concluded that cortical abnormalities are an age-dependent endophenotype affected by genetic or symptom burden in siblings of schizophrenia patients.^15^ Particularly, with increased genetic burden, grey matter abnormalities in siblings are progressive in adulthood. Therefore, determining the relationship between genotypes and phenotypes in schizophrenia patients and their unaffected siblings can provide deeper insights into the genetic mechanisms underlying schizophrenia.^16^

In the current study, we estimated magnetic resonance imaging (MRI)-derived cortical thickness across multiple brain regions in three groups: schizophrenia patients (SZ), unaffected siblings of patients (SB), and healthy controls (HC). We first identified the between-group variation in cortical thickness and genetic variants of target regions (108 independent loci from the Psychiatric Genomics Consortium findings). We then studied the association between cortical thickness in abnormal regions in SZ and genetic risk variants. Finally, we explored the phenotypic differences between groups with different genetic bases. Our aim was to identify genetic risk variants that influence cortical thickness in schizophrenia and their effects on phenotype, which is indispensable to understanding the pathogenesis of schizophrenia.

## Methods

### Participants

For this study, we recruited 129 first-episode patients diagnosed with schizophrenia from the inpatient and outpatient clinics of the Second Xiangya Hospital Institute of Mental Health and 42 of their SB. A total of 112 HC were recruited from the community, hospital staff, and schools. Patients were interviewed using the patient version of the Structured Clinical Interview for DSM-IV.^17^ Schizophrenia was diagnosed according to DSM-IV criteria by trained psychiatrists, and the diagnosis did not change during ≥ 6 months of follow-up. SB and HC were screened with the non-patient version of the same test to ensure they did not meet the diagnosis criteria of any mental disorder. All participants were right-handed, native Chinese, and aged between 12 and 40 years. Further details about the sample can be found in previous publications.^16^,^18^

Participants were excluded if they had any of following conditions: 1) substance-related disorders; 2) any reported history of substance-related disorders, neurological disorders, syncope, or serious physical illness, either personally or among first-degree relatives; 3) intellectual disability (intelligence quotient < 70) or significant intellectual disability; and 4) previous electroconvulsive therapy or any contraindication for MRI. This study was approved by the institutional review broad of the Second Xiangya Hospital of Central South University. All data collection and analysis procedures were in strict accordance with relevant guidelines and regulations. Informed written consent was obtained from all participants after the study’s purpose and procedures were explained.

### Clinical and cognitive assessment

This study’s clinical evaluation included the following aspects: symptom burden, dosage and duration of medication, duration of disease, onset form, and cognitive performance. Symptom severity was evaluated through the Positive and Negative Syndrome Scale^19^ and the Schizophrenia Suicide Risk Scale.^20^ Functional outcome was measured using the Social and Occupational Functional Assessment Score.^21^ Antipsychotic load (converted into chlorpromazine equivalents)^22^ and duration of psychotic medication were determined. Symptom onset was calculated from the date of the first noticeable psychotic symptoms (delusions, hallucinations, or disorganized speech/behavior) reported by self or family, and illness duration was determined from the symptom onset date. Onset type was classified as acute (1-7 days), subacute (1-4 weeks), or gradual (> 1 month).^23^

Cognition was assessed through six cognitive tasks^16^: the N-back task as a measure of working memory, the logical memory test as a measure of verbal declarative memory, the digit span test as a measure of numerical memory, the visual patterns test as a measure of visual recall (see Figure S1, available on the online-only supplementary material), the verbal fluency test as a measure of language function, a series of arithmetic problems as a measure of computational function, and a modified Wisconsin card sorting test as a measure of cognitive transfer ability and overall cognitive level. A detailed description of these tasks is provided in Supplementary Material S1 (available online only).

To reduce multiple comparisons, principal component analysis (PCA) was applied across the whole patient group by extracting components that accounted for the majority of variance for each cognitive task.^6^ For the N-back task, target accuracy was 0 back, 2 back, and for the whole test, in addition to N-back non-target accuracy (whole test error rate) were entered into the PCA; for the logical memory test, the number of immediate and delayed correct responses were entered into the PCA; for digit span test, the number of sequential correct responses and the number of inverted correct responses were entered into the PCA; for the visual patterns test, the number of correct responses and level of highest completed difficulty were entered into the PCA. One principal component was extracted for all these tasks (which, separately, accounted for 61.9, 91.1, 69.6, and 75.1% of the variance).

### Magnetic resonance imaging data acquisition and preprocessing

On the same day as the clinical and cognitive assessment, all participants underwent structural MRI scanning using the same 3.0-T Magnetom Skyra scanner (Siemens Healthineers, Erlangen, Germany), which is equipped with a standard head coil for radio frequency transmission and reception of nuclear MR signals. Participants were instructed to keep their eyes closed, move as little as possible and not think about any particular thing. Foam pads and earplugs were used to minimize scanner noise and head motion. The structural MRI images were acquired with a three-dimensional T1-weighted magnetization-prepared rapid acquisition gradient echo sequence, and scanner parameters were repetition time = 1,900 ms, echo time = 2.01 ms, inversion time = 900 ms, flip angle = 9°, slices = 176, matrix size = 256 × 256, contiguous slices with slice thickness = 1.0 mm, and field of view = 256 × 256 mm^2^.

With FreeSurfer software (http://surfer.nmr.harvard.edu, version 6.0.0), a surface-based approach was used to calculate whole-brain cortical thickness. Following skull-stripping and intensity correction, the gray/white matter boundary for each cortical hemisphere was determined through tissue intensity and local constraints. The resulting surface boundary was tessellated to produce multiple vertices across the whole brain before inflating. Using a deformable surface algorithm guided by the gray matter/cerebrospinal fluid intensity gradient, the resulting gray/white matter interface was expanded to create the pial surface. The inflated surface was then morphed into a sphere, followed by registration to an average spherical surface for optimal sulcogyral alignment. After the above procedures, the Desikan-Killiany Atlas (68 regions) was used to extract the cortical thickness of each region with FreeSurfer. Topological defects were corrected manually by two members of the research staff.

### Targeted gene selection and sequencing

Since the samples were collected and sequenced in 2014, the target genes include upstream and downstream variants of 1mb in 108 schizophrenia risk loci (128 genes) identified in a recent genome-wide association study by the Schizophrenia Working Group’s Psychiatric Genomics Consortium.^2^ For each of these genes, both coding and non-coding (regulatory) regions were included in the sequencing target. The regulatory genomic regions consisted of a 5′untranslated region, a 3′untranslated region, and intron-exon boundaries (25 bp). Custom capture oligos were designed using the SureDesign website (Agilent Technologies, Santa Clara, CA, USA).

Genomic DNA was extracted from 2 mL of ethylene diamine tetra acetic acid from anticoagulated peripheral blood, fragmented, and used for sample library construction (HiSeq, Illumina, San Diego, CA, USA) according to manufacturer instructions. Briefly put, genomic DNA was fragmented to a pool of 100-500 bp, and then adapters (Invitrogen, Carlsbad, CA, USA) were ligated to both ends of the resultant fragments. The adapter-ligated templates were purified using MagPure A3 XP beads (Beckman Coulter, Brea, CA, USA). The purified DNA was amplified by ligation-mediated polymerase chain reaction, purified, and hybridized to library fragments. The hybridized fragments were bound to Streptavidin Dynabeads (Invitrogen) and washed with stringent buffers. The captured products were quantified using a Qubit dsDNA HS Assay Kit (Invitrogen). Paired-end sequencing, which reads 150 bases from each end of the fragment for targeted libraries, was performed using Illumina HiSeq Xten and NovaSeq instrumentation (Illumina). Information on target sequencing region and sequencing quality of all samples are shown in Tables S1 and S2, available as online-only supplementary materials (Excel files for download).

### Statistical analysis

#### Comparison of demographic, clinical, and cognitive performance among groups

Inter-group comparison and correlation analysis were performed in IBM SPSS Statistics (IBM, Armonk, NY, USA). One-way analysis of variance and chi-square analysis were used to compare demographical, clinical, and cognitive indices among the SZ, SB, and HC groups.

#### Comparison of cortical thickness among groups

A general linear model was used to determine differences in cortical thickness among groups, with age and sex as covariates. The false discovery rate was applied to correct the multiple comparisons.

#### Comparison of single nucleotide variants in high-risk genes and polygenic risk scores

The chi-square test was used to analyze the associations between single nucleotide polymorphisms and diagnosis by PLINK (https://zzz.bwh.harvard.edu/plink/).^24^ In this study, we used Fisher’s exact test throughout to avoid bias due to distributional approximation. Based on the association analyses in target genes, the PRS of target regions were constructed using the PRSice toolbox.^25^ A detailed description of PRS calculation is provided in Supplementary Material S1.

#### Correlation and subgroup analyses

We conducted a correlation analysis between PRS and cortical thickness or cognitive indices in all participants. Especially in SZ, we conducted a correlation analysis between the cortical thickness of brain regions with significant SNVs and cognitive indices. We compared demographic and clinical differences between gene subgroups of patients using two-sample *t*-tests and chi-square analysis. Analysis of covariance was used for cognitive indices among gene subgroups of patients, controlling for age and sex as covariates.

## Results

### Participant characteristics

A total of 299 patients were recruited for this study, of whom 16 failed to complete clinical data acquisition and MRI scanning (due to poor quality results). Thus, the final sample included 283 participants (129 in the SZ group, 42 in the SB group, and 112 in the HC group) who completed the following steps: general information collection, clinical and cognitive assessment, and MRI scanning. Of these, high-quality genotyping in target region (5,079 single nucleotide polymorphisms) was performed for 263 (116 SZ, 41 SB and 106 HC). The demographic, clinical, and cognitive characteristics of the participants are shown in Table 1. Significant age differences (p < 0.001) were found among the groups. As expected, SZ performed worse in all cognitive tests than SB or HC (Table 1). SB performed worse in the logical memory (p < 0.001) and visual memory (p < 0.001) tests than HC (Table S3, available as online-only supplementary material).

### Cortical thickness features among the groups

**Figure 1:**
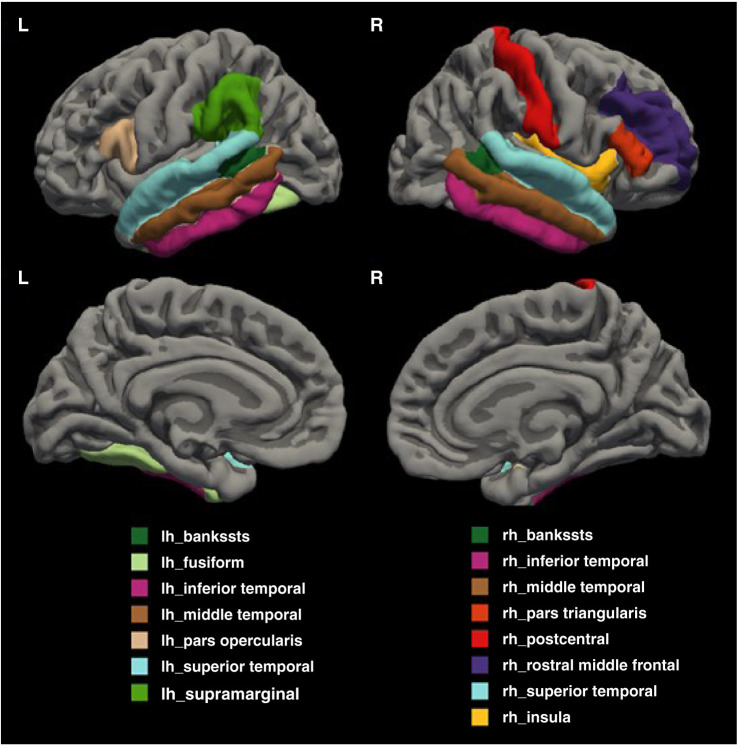
Illustrations of the 15 brain regions that differed among schizophrenia patients, healthy controls, and unaffected siblings. bankssts: banks of the superior temporal sulcus; lh: left hemisphere; rh: right hemisphere.

A general linear model with age and sex as covariates revealed significant cortical thickness differences among the groups in 15 of the 68 regions (as shown in Figure 1) of the whole brain, including the bilateral banks of the superior temporal sulcus, bilateral inferior temporal, bilateral middle temporal, bilateral superior temporal, left fusiform, left pars opercularis, left supramarginal, right pars triangularis, right postcentral, right rostral middle frontal, and right insula. The multiple comparison post hoc test is shown in Table S4 (available as online-only supplementary material). In these regions, SZ had a thinner cortex than HC, although SB did not, except for the left middle temporal region.

### Single nucleotide variants and polygenic risk score results

Summary data on association analysis and single nucleotide polymorphisms for PRS calculation in the final model are shown in Tables S5 and S6, available as online-only supplementary materials (Excel files for download). Association analyses in target genes found four SNVs that were significantly associated with schizophrenia diagnosis after correction for false discovery rate (FDR): SNV20673 in thioredoxin-related transmembrane protein 2 (TMX2)-catenin, cadherin-associated protein, delta 1 (CTNND1) gene (p-value after FDR correction [P_FDR_] = 0.008), rs35542507, rs41277477, and rs73165153 in the centromere protein M gene (P_FDR_ = 0.030). In our sample, rs35542507, rs41277477, and rs73165153 were observed in a complete linkage state in linkage disequilibrium analysis. The Manhattan plot of association analyses in target genes is shown in Figure S2, available as online-only supplementary material.

To construct the PRS, we obtained the optimal model through threshold calculation (Figure S3), which contained a total of 702 single nucleotide polymorphisms. There were significant differences among the three groups (PRS_SZ_ = 0.00682±0.00362, PRS_HC_ = -0.00498±0.00339, and PRS_SB_ = 0.00767±0.00412). There were significant negative correlations between PRS and cortical thickness in the left middle temporal, pars opercularis, superior temporal, supramarginal, right post-central, rostral middle frontal, and superior temporal regions, with age, sex, and group as covariates in the whole sample (Table S7).

### The association between single nucleotide variants and cortical thickness

**Figure 2:**
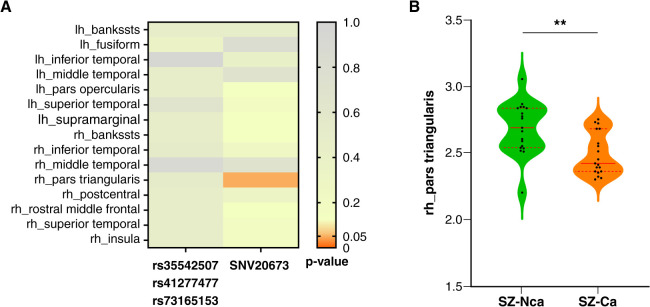
A) Heat map of associations between SNV20673, rs35542507, rs41277477, rs73165153, and cortical thickness in 15 regions of interest. B) Cortical thickness differences in right pars triangularis between schizophrenia patients who are carriers and non-carriers of SNV20673. bankssts: banks of the superior temporal sulcus; lh = left hemisphere; rh = right hemisphere; SNV = single nucleotide variants; SZ-Ca: schizophrenia group SNV20673 carriers; SZ-Nca: schizophrenia group SNV20673 non-carriers. ** p < 0.01.

In regions with group effects, after multivariate analysis with age and sex as covariates, rs35542507, rs41277477, and rs73165153 had no main effects on cortical thickness, but SNV20673 had a significant main effect on cortical thickness in the right pars triangularis in the whole sample (Figure 2A). Carriers of the SNV20673 risk allele had significantly lower (P_FDR_ = 0.048) cortical thickness in the right pars triangularis than non-carriers. There were no significant differences between SZ and SB for SNV20673 (p = 0.793), rs35542507, rs41277477, or rs73165153 (p = 0.479). In SZ, to minimize the impact of potential confounding variables and quantity imbalances, we compared age- (p = 0.999) and sex-matched (p = 0.999) non-carriers (n = 19/97) with carriers (n = 19/19). The carriers of the SNV20673 risk allele also showed significantly lower cortical thickness in the right pars triangularis than non-carriers (P_FDR_ = 0.013) (Figure 2B).

### Differences between patients with and without the SNV20673 risk allele

**Figure 3:**
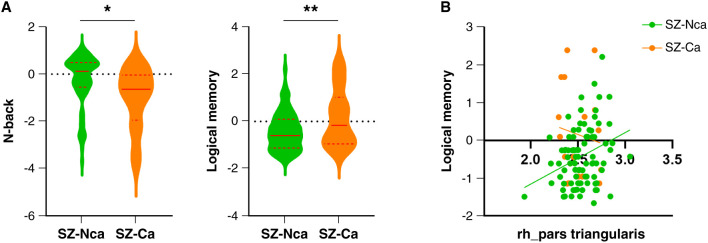
A) Comparisons of logical memory and N-back test results between schizophrenia patients according to SNV20673 carrier status. B) Correlation between logical memory test and right pars triangularis thickness in schizophrenia patients according to SNV20673 carrier status. rh: right hemisphere; SZ-Ca: schizophrenia group SNV20673 carriers; SZ-Nca: schizophrenia group SNV20673 non-carriers. * p < 0.05; ** p < 0.01.

In SZ, clinical symptoms did not differ significantly between carriers and non-carriers of the SNV20673 risk allele, although carriers had a more urgent (p = 0.030) illness onset (Table S8 and Figure S4B) than non-carriers. There were significant differences in cognitive characteristics between carriers and non-carriers in the N-back and logical memory tests (Figure 3A). Carriers had worse performance in the N-back task (p = 0.022) but better performance in the logical memory test (p = 0.007) than non-carriers, which was significantly correlated (Spearman’s rho = 0.199, p = 0.032) with cortical thickness in the right pars triangularis (Figure 3B).

There were significant differences in arithmetic (p = 0.027), logical memory (p < 0.001), and visual memory (p = 0.002) performance among SB group SNV20673 carriers and non-carriers and HC group SNV20673 non-carriers (Figure S4A).

## Discussion

The current study examined group effects (SZ, SB, and HC) on cortical thickness and genetic variants of 108 independent loci found by PGC, exploring the relationship among diagnosis groups, cortical thickness, and genetic variants and finding significant diagnostic effects. There were three main findings: first, the association analysis in target genes between SZ and HC found four variants with significant group effects in TMX2-CTNND1 (SNV20673) and the centromere protein M (rs35542507, rs41277477, rs73165153) gene; second, carriers of the SNV20673 variant had lower cortical thickness than non-carriers; third, the bilateral temporal, frontal, and parietal cortex of SZ was thinner than HC, while the cortical thickness of SB was between that of SZ and HC in most of these regions. However, the differences between SB and either SZ or HC did not survive correction for multiple testing. A positive correlation was also found between right pars triangularis thickness and logical memory test performance in SZ. To the best of our knowledge, this is the first study to assess cortical thickness and polymorphism in schizophrenia patients and their unaffected siblings, investigating the relationship between cortical alterations, genetic risks, and clinical symptoms.

The effort to uncover schizophrenia risk genes has been expanding for many years, and an increasing number of candidates have been identified.^26^ PRS for schizophrenia have also been calculated to evaluate the total genetic burden of schizophrenia on an individual level.^27^ The association between PRS for schizophrenia and cortical thickness has been widely reported in healthy individuals^11^ and schizophrenia patients.^28^ We consistently found that PRS were negatively associated with cortical thickness in several brain regions. However, across these PRS studies, the SNV sets included in PRS calculation did not entirely overlap. Thus, it is difficult to locate specific molecular pathways according to PRS^29^; subsequent candidate gene analysis is necessary.

Unlike PRS, candidate gene analysis reflected the pathway effect of SNVs and specifically related brain regions. We observed two genes associated with schizophrenia: TMX2-CTNND1 (SNV20673) and CTNND1 (rs35542507, rs41277477, and rs73165153). These two genes have been found repeatedly in genome-wide association studies^2^,^3^ in both Asian and European populations.^4^ TMX2-CTNND1 is a naturally occurring read-through transcription gene between the neighboring TMX2 and CTNND1 genes on chromosome 11. CTNND1 was found to be significantly associated with schizophrenia in proteome-wide analysis.^30^ Intriguingly, CTNND1 variants disrupt the cadherin/catenin complex and inactivate Wnt signaling activity,^31^ which regulates cortical size by controlling whether progenitors continue to proliferate or exit the cell cycle to differentiate.^32^ TMX2, a sensor in the mitochondria-associated membrane-regulated redox signaling pathway, was identified as a key adaptive regulator of neuronal proliferation, migration, and organization in the developing brain.^33^ Our findings provide further insight into the mechanism of schizophrenia, especially the calcium channel and redox dysregulation hypotheses.

Specifically, the present study revealed the effect of SNV20673 (in TMX2-CTNND1) on cortical thickness in the right pars triangularis. The pars triangularis is located in the inferior frontal gyrus and was formerly known by its functional and cytoarchitectural title, Brodmann’s area 45, which encompasses Broca’s area and Brodmann’s area 44 (pars opercularis).^34^ It has been demonstrated that Broca’s area is involved in expressive aspects of spoken and written language. Many studies have reported structural atrophy and impaired functional connectivity in Broca’s area in schizophrenia patients and their relatives.^35^-^37^ Interestingly, Zhang et al.^37^ reported that cortical thickness in the bilateral ventromedial prefrontal cortices, left superior temporal gyrus, and right pars triangularis was lower in schizophrenia patients with untreated chronic illness than in healthy controls. In never-medicated patients, the influence of antipsychotics was ruled out, which suggests that right pars triangularis cortical thickness may be a trait marker for schizophrenia. On a micro level, Makowski et al.^9^ revealed that differential methylation and human-specific single nucleotide polymorphisms were associated with perisylvian thickness (including language areas such as Broca’s area). The present study also found a significant positive correlation between cortical thickness in the right pars triangularis and logical memory performance in SZ. Similarly, a previous study found that schizophrenia patients with a thinner right pars triangularis gyrus cortex may be more prone to cognitive deterioration.^36^ A longitudinal study found that verbal memory improvement was associated with increased baseline cortical thinning in the left pars triangularis in first-episode schizophrenia.^38^ Our findings extend the evidence base by showing that variation in SNV20673 and TMX2-CTNND1 is associated with changes in cortical thickness in the right pars triangularis, influencing the association between cortical thickness and cognitive function in schizophrenia. Although our sample size was relatively small for a genetics study, the MRI sample could be considered midsized.^39^ We also applied relatively strict correction to ensure sufficient statistical power. We believe that our research represents a meaningful contribution to understanding the mechanisms of schizophrenia.

In recent years, cortical thinning of specific brain regions has emerged as a consistent feature of schizophrenia, especially in the frontal and temporal lobes.^7^ Consistent with these findings, cortical thickness was lower in most bilateral temporal lobes, Broca’s areas, left face recognition areas, and right motor areas in SZ than HC. Surprisingly, the corrected results showed no significant differences between SB, SZ, and HC, although the latter two differed significantly. These findings indicate that the cortical thickness of SB is likely somewhere between that of SZ and HC. According to the uncorrected inter-group results and mean cortical thickness in these regions with respect to diagnostic effect (Table S4), cortical thickness was lower in SZ than SB in some temporal regions, except the left pars opercularis, which was higher in SB. A previous study also found that schizophrenia patients had thinner frontal and temporal cortices than healthy controls, although the results were less pronounced when patients were compared with their non-psychotic relatives.^40^ In its most rudimentary form, patients and their unaffected siblings can be understood as being in the onset and susceptibility stages, respectively, which might explain their morphological differences. Moreover, in both SZ and SB, we found that the right pars triangularis cortex was thinner in SNV20673 carriers than non-carriers. Thus, our findings further support the hypothesis that gene pathways could substantially contribute to abnormal endophenotypes in schizophrenia. We also found that the left middle temporal cortex was thinner in SB than HC. Consistent with our findings, Honea et al.^41^ also found lower temporal gray matter volumes in siblings of schizophrenia patients than controls. Studies that included first-degree relatives of schizophrenia patients and youth at high familial risk of schizophrenia reported lower cortical thickness in the left and right middle temporal gyrus.^42^,^43^ The differences between the SB and HC groups in our study could have been related to genetic vulnerability, which may not directly contribute to symptoms.

Our study involved several limitations that should be mentioned. First, we did not obtain enough SNV20673 carriers in the HC group (only one); a larger sample is needed to clarify the effects of SNVs in healthy populations. Second, since most of our patients were being treated with antipsychotics, we urge caution when generalizing these findings to untreated cohorts. Third, we only used cortical thickness and the Desikan-Killiany Atlas to explore cortical phenotypes; future studies should include the cortical surface area and local gyrification index, focusing on language-related areas. Finally, although we used age as a covariate in the analysis model, the effect of age on morphology cannot be completely ruled out. Post-hoc analysis in the adult sample is provided in Supplementary Material S2. Detailed demographic, clinical, and cognitive characteristics of the adult participants are shown in Table S9. Post-hoc analysis in adult sample also showed a significant main effect on cortical thickness in the left pars triangularis (P_FDR_ = 0.016) (Figure S5). Additionally, we did not control for education level in this study, especially since educational deficits are inherent to schizophrenia *per se*, thus adjusting for it may remove some crucial disease-related variance. Gene expression and translation, as well as longitudinal changes, warrant further research. The present study provides clues about the link between genes, the cortex, and disease.

In conclusion, based on cortical morphology and schizophrenia candidate gene loci, the present study revealed an association between candidate gene locus and cortical thickness, as well as between cortical thickness, diagnosis, and phenotypic traits, especially the effects of SNV20673 in TMX2-CTNND1 on the right homologue of Broca’s area, which contributed to the pathogenesis of schizophrenia. Our findings provide evidence that genetic factors cause schizophrenia susceptibility or onset by influencing brain structure, which furthers our understanding of the disease’s mechanism.

## Disclosure

The authors report no conflicts of interests.

## Figures and Tables

**Table 1:** Detailed demographic, clinical, and cognitive characteristics of the participants

	SZ (n = 129)	HC (n = 112)	SB (n = 42)	F/χ^2^	p-value
Age (years)	18.03±3.27	21.72±3.88	22.24±6.02	32.1	< 0.001
Sex	69/60	58/54	14/28	5.4	0.066
N-back†	-0.36±1.13	0.41±0.56	0.14±1.03	16.5	< 0.001
Logical memory†	-0.32±0.89	0.50±0.98	-0.17±0.91	20.6	< 0.001
Digit span†	-0.30±0.99	0.42±0.91	0.03±0.90	15.1	< 0.001
Visual memory†	-0.32±0.80	0.56±1.11	-0.23±0.77	25.8	< 0.001
Arithmetic	14.06±4.36	17.34±3.85	15.70±4.22	16.3	< 0.001
Verbal fluency	17.23±5.65	21.91±6.28	20.13±4.96	17.6	< 0.001
MWCST	29.98±8.58	37.92±6.28	34.97±8.54	26.9	< 0.001
SOFAS	62.94±15.56	93.55±4.27	91.15±6.74	214.1	< 0.001
DoI (months)	21.71±24.44	-	-	-	-
DoM (months)	13.28±21.45	-	-	-	-
CPZ equivalents (×100 mg/d)	2.33±1.61	-	-	-	-
PANSS	70.40±24.53	-	-	-	-
SSRS	14.29±8.29	-	-	-	-

CPZ = chlorpromazine; DoI = duration of illness; DoM = duration of medication; HC = healthy controls; MWCST = Modified Wisconsin Card Sorting Test; PANSS = Positive and Negative Syndrome Scale; SB = unaffected siblings; SOFAS = the Social and Occupational Functioning Assessment Scale; SSRS = Schizophrenia Suicide Risk Scale; SZ = schizophrenia patients.

† Principal component analysis.

## Data Availability

The data that support the findings of this study are available from the corresponding author upon reasonable request.
