# Supplementary material for: Influence of TMX2-CTNND1 polymorphism on cortical thickness in schizophrenia patients and unaffected siblings: an exploratory study based on target region sequencing
Source: Braz J Psychiatry. 2024 Mar 25;46:e20233322. doi: 10.47626/1516-4446-2023-3322 (PMC11189138; doi:10.47626/1516-4446-2023-3322)
Supplement: Supplementary file 1 [file bjp-46-e20233322-s001.pdf]

Supplementary Material S1

Cognitive assessment

The N-back task has been extensively used in previous studies by our research group. All participants performed a parametric N-back task on Nordic Neurolab's fMRI hardware system for 8 minutes and 16 seconds. The stimuli were sequences of white capital letters on a black background, presented centrally (500 ms duration, 1500 ms inter-stimulus interval) in a pseudo-random order. Task performance, represented by the reaction time and accuracy of each participant, was recorded electronically.

The logical memory test, the digit span task, the arithmetic test, and the verbal fluency test were based on the revised Chinese version of Wechsler's intelligence scale. The logical memory test consists of a brief story that is presented for two recall trials (immediately and 30 min later). The digit span test is a verbal memory test. The participant is instructed to recall the sequence of numerical digits to the experimenter after hearing them. The sequence becomes increasingly longer in each trial. The test ends when the participant cannot accurately recall the full sequence of digits. The participant's score is the longest number of sequential digits that can accurately be remembered. The digit span test can also be given backward, meaning that the participant is asked to recall the sequence in reverse order. The arithmetic test consists of 20 mental addition, subtraction, multiplication and division problems. Verbal fluency was tested by asking participants to report as many animals as possible in 60 s. Due to time constraints, we did not conduct a complete set of Wechsler intelligence tests. We converted the total score of the scales we used into a hundred-point system to screen for severe intellectual impairment during the recruitment phase.

The visual patterns test, a highly reliable measure of visual memory, was used to assess visual working memory. Participants were shown a series of stimulus cards (each for 3 s) with various patterns of black and white squares in a checkerboard grid and were then asked to reproduce the patterns by marking only the black squares on a blank grid. The test consists of 14 levels of increasing complexity (three patterns per level, 42 in total). The highest completed level was considered the performance score. Two alternative versions of the test were available to minimize the practice effect.

Figure S1 Examples of simple and complex matrix patterns in the visual patterns test[1]

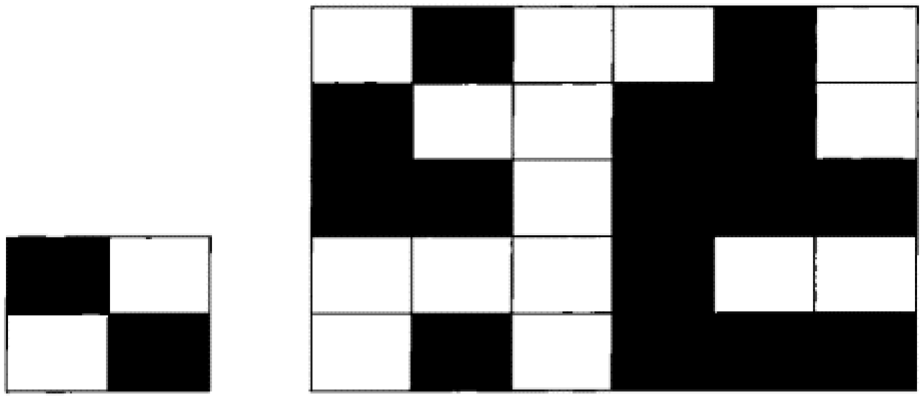

The Wisconsin card sorting test measures the ability to develop new concepts and shift sets. The test consists of 4 stimulus cards, each of which is unique in terms of color (red, green, blue, or yellow), shape (triangle, star, cross, or circle), and number of items (one, two, three, or four). The participant is required to place each response card below a stimulus card in the best-fitting position. After each response the participant is told whether this was right or wrong, with no other cues given at any stage. The modified Wisconsin test uses the same stimulus cards, but all cards sharing more than one attribute with a stimulus card (for example, the same color and shape) are eliminated, leaving a 24-card set, which is then duplicated. Overall proficiency on the test was determined by the number of categories achieved (maximum 6) and the total number of errors made. In patients who failed to complete the test, an additional analysis of the error type (perseverative or non-perseverative) was performed.

Polygenic risk score calculation

The polygenic risk score (PRS) was calculated through association analysis in PLINK[2] for single nucleotide polymorphisms (SNP) and diagnosis using the χ^2 test. Fisher's exact test was then used throughout to avoid bias due to distributional approximation. Based on the association analyses in target genes, the PRS of target regions was constructed using the PRSice toolbox.[3] The basic formula was PRS = sum(lnORxSNP)/N, in which the odds ratio (OR) of each SNP was defined by the above-mentioned association analysis, SNP represents the genotype of each sample, and N represents the number of SNPs in the model. As shown in Figure S2, we obtained the optimal model through threshold calculation, which contains a total of 702 SNPs, and we calculated the PRS of each sample.

Table S1 and **Table S2** are available as Excel files for download.

Table S3 Multiple comparisons among schizophrenia patients, unaffected siblings, and healthy controls regarding demographic and cognitive characteristics

	SZ (mean ± SD)	SB (mean ± SD)	HC (mean ± SD)	P-value		
				SZvsHC	SBvsHC	SZvsSB
Age (years)	18.03±3.27	22.24±6.02	21.72±3.88	<0.001	0.864	**<0.001**
Arithmetic	-0.36±1.13	0.14±1.03	0.41±0.56	<0.001	0.100	0.079
Verbal fluency	-0.32±0.89	-0.17±0.91	0.50±0.98	<0.001	0.239	**0.017**
MWCST	-0.30±0.99	0.03±0.90	0.42±0.91	<0.001	0.163	**0.010**
N-back_PCA	-0.32±0.80	-0.23±0.77	0.56±1.11	<0.001	0.311	0.044
Logical_Memory_PCA	14.06±4.36	15.70±4.22	17.34±3.85	<0.001	**0.001**	0.666
Number_Memory_PCA	17.23±5.65	20.13±4.96	21.91±6.28	<0.001	0.084	0.134
Visual_Memory_PCA	29.98±8.58	34.97±8.54	37.92±6.28	<0.001	**<0.001**	0.802
SOFAS	62.94±15.56	91.15±6.74	93.55±4.27	<0.001	0.105	**<0.001**

HC= healthy controls; MWCST = modified Wisconsin card sorting test; PCA = principal component analysis; SB = unaffected patient siblings; SOFAS = Social and Occupational Functioning Assessment Scale; SZ = schizophrenia patients.

Table S4 Multiple comparisons among groups regarding cortical thickness and diagnostic effects

	SZ	SB	HC	P_{SZvsHC}	P_{SBvsHC}	P_{SZvsSB}
lh_bankssts_thickness	2.424±0.181	2.459±0.156	2.469±0.161	**0.001**	0.662	**0.034**
lh_fusiform_thickness	2.725±0.145	2.759±0.152	2.757±0.121	**0.009**	0.685	**0.010**
lh_inferiortemporal_thickness	2.831±0.171	2.861±0.191	2.863±0.120	**0.006**	0.829	**0.019**
lh_middletemporal_thickness	2.835±0.155	2.826±0.145	2.885±0.152	**<0.001**	**0.039**	0.149
lh_parsopercularis_thickness	2.641±0.156	2.637±0.171	2.685±0.126	**<0.001**	0.106	**0.014**
lh_superiortemporal_thickness	2.797±0.191	2.807±0.154	2.841±0.154	**0.002**	0.241	0.275
lh_supramarginal_thickness	2.520±0.153	2.536±0.175	2.555±0.141	**0.002**	0.499	**0.041**
rh_bankssts_thickness	2.555±0.180	2.612±0.188	2.594±0.184	**0.001**	0.588	**0.002**
rh_inferiortemporal_thickness	2.864±0.152	2.889±0.148	2.897±0.134	**0.006**	0.991	**0.013**
rh_middletemporal_thickness	2.870±0.155	2.893±0.149	2.910±0.145	**<0.001**	0.742	**0.009**
rh_parstriangularis_thickness	2.535±0.167	2.549±0.153	2.576±0.143	**<0.001**	0.311	0.078
rh_postcentral_thickness	2.047±0.123	2.066±0.122	2.092±0.114	**0.004**	0.193	0.219
rh_rostralmiddlefrontal_thickness	2.400±0.130	2.403±0.112	2.428±0.112	**0.006**	0.298	0.162
rh_superiortemporal_thickness	2.851±0.172	2.886±0.139	2.895±0.151	**0.001**	0.674	**0.034**
rh_insula_thickness	3.000±0.144	2.988±0.132	3.035±0.162	**0.001**	0.293	0.438

bankssts = banks of the superior temporal sulcus; HC = healthy controls; SB = unaffected patient siblings; SZ = schizophrenia patients.

http://doi.org/10.47626/1516-4446-2023-3322

Table S5 and **Table S6** are available as Excel files for download.

Figure S2 The Manhattan plot of target-region genome-wide association studies.

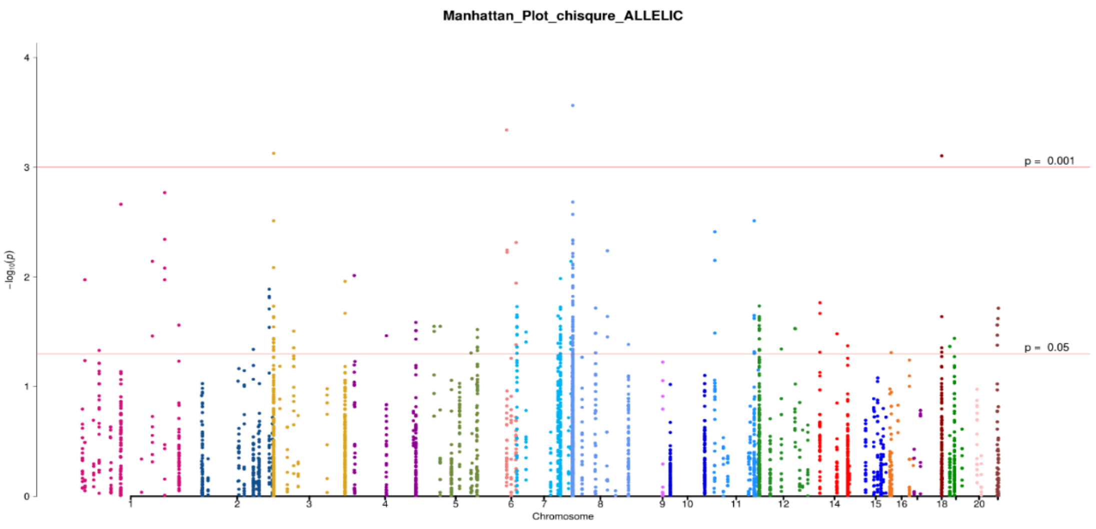

Figure S3 R^2 and p-values of the PRS model with different thresholds

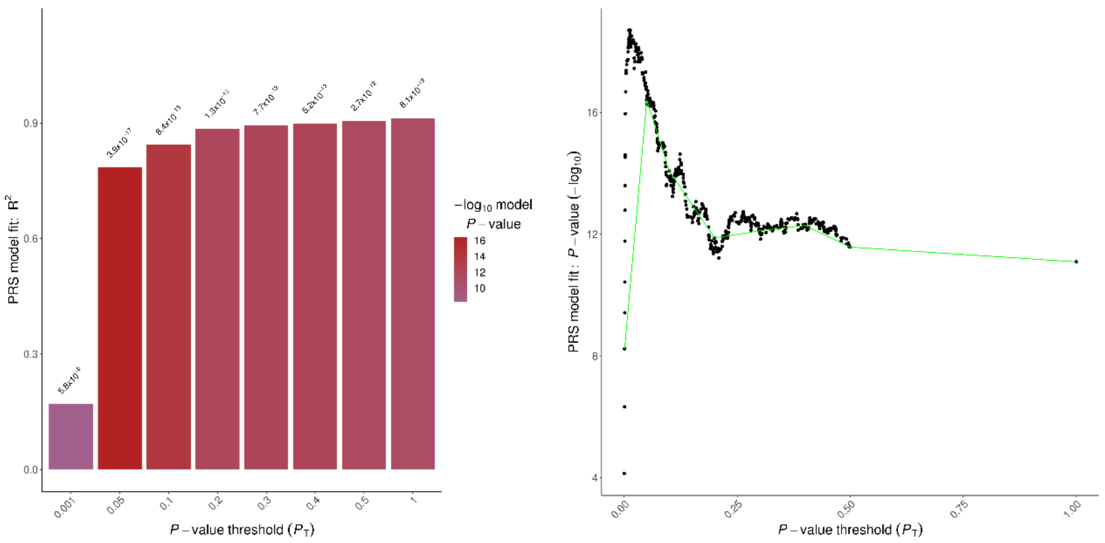

http://doi.org/10.47626/1516-4446-2023-3322

Table S7 Correlation coefficients between polygenic risk scores and cortical thickness of each brain region in the whole sample

	r	P	P_{FDR}
lh_middletemporal	-0.197	0.001	0.005
lh_parsopercularis	-0.231	<0.001	0.003
lh_superiortemporal	-0.160	0.010	0.018
lh_supramarginal	-0.171	0.006	0.012
rh_middletemporal	-0.135	0.029	0.048
rh_postcentral	-0.201	0.001	0.005
rh_rostralmiddlefrontal	-0.183	0.003	0.008
rh_superiortemporal	-0.181	0.003	0.008
rh_insula	-0.199	0.001	0.005

Table S8 Detailed demographic, clinical, and cognitive characteristics of the patients according to SNV20673 carrier status

	**SZ-Nca (n=97)**	**SZ-Car (n=19)**	**t/χ2/F**	**p-value**
Age, years	17.91±3.36	18.74±3.51	-0.976	0.331
Sex	53/44	9/10	0.338	0.561
PANSS	68.64±24.64	73.05±22.66	-0.723	0.471
PANSS-N	17.66±10.34	17.58±7.21	0.041	0.967
PANSS-P	16.69±8.02	18.37±7.40	-0.844	0.400
PANSS-G	30.52±10.72	31.84±10.57	-0.495	0.622
SSRS	14.12±7.59	11.63±9.18	1.263	0.209
Mode of onset (acute/sub-acute/gradual)	17/9/69	8/3/8	7.016	0.030
DoI (months)	21.86±25.02	20.05±26.85	0.284	0.777
DoM (months)	14.25±23.37	10.09±14.97	0.745	0.789
CPZ equivalents (×100mg/d)	2.26±1.62	2.87±1.45	-1.534	0.458
SOFAS	63.36±16.01	62.42±15.67	0.235	0.815
N-back_PCA	-0.24±1.09	-0.99±1.29	5.420	0.022
Logical_Memory_ PCA	-0.41±0.79	0.17±1.22	7.515	0.007
Number_Memory_ PCA	-0.36±0.97	0.02±0.74	2.183	0.142
Visual_Memory_ PCA	-0.27±0.71	-0.45±0.88	0.665	0.417
Arithmetic	14.57±4.35	13.21±3.55	1.986	0.162
Verbal fluency	17.05±5.18	18.58±6.66	1.224	0.271
MWCST	29.90±8.79	31.13±7.96	0.173	0.678

CPZ = chlorpromazine; DoI = duration of illness; DoM = duration of medication; MWCST = modified Wisconsin card sorting test; PANSS = the Positive and Negative Syndrome Scale; PCA = principal component analysis; SOFAS = the Social and Occupational Functioning Assessment Scale; SSRS = the Schizophrenia Suicide Risk Scale; SZ-Ca = schizophrenia group SNV20673 carriers; SZ-Nca = Schizophrenia group SNV20673 non-carriers.

http://doi.org/10.47626/1516-4446-2023-3322

Figure S4 Cognitive and onset characteristics among diagnosis × gene groups

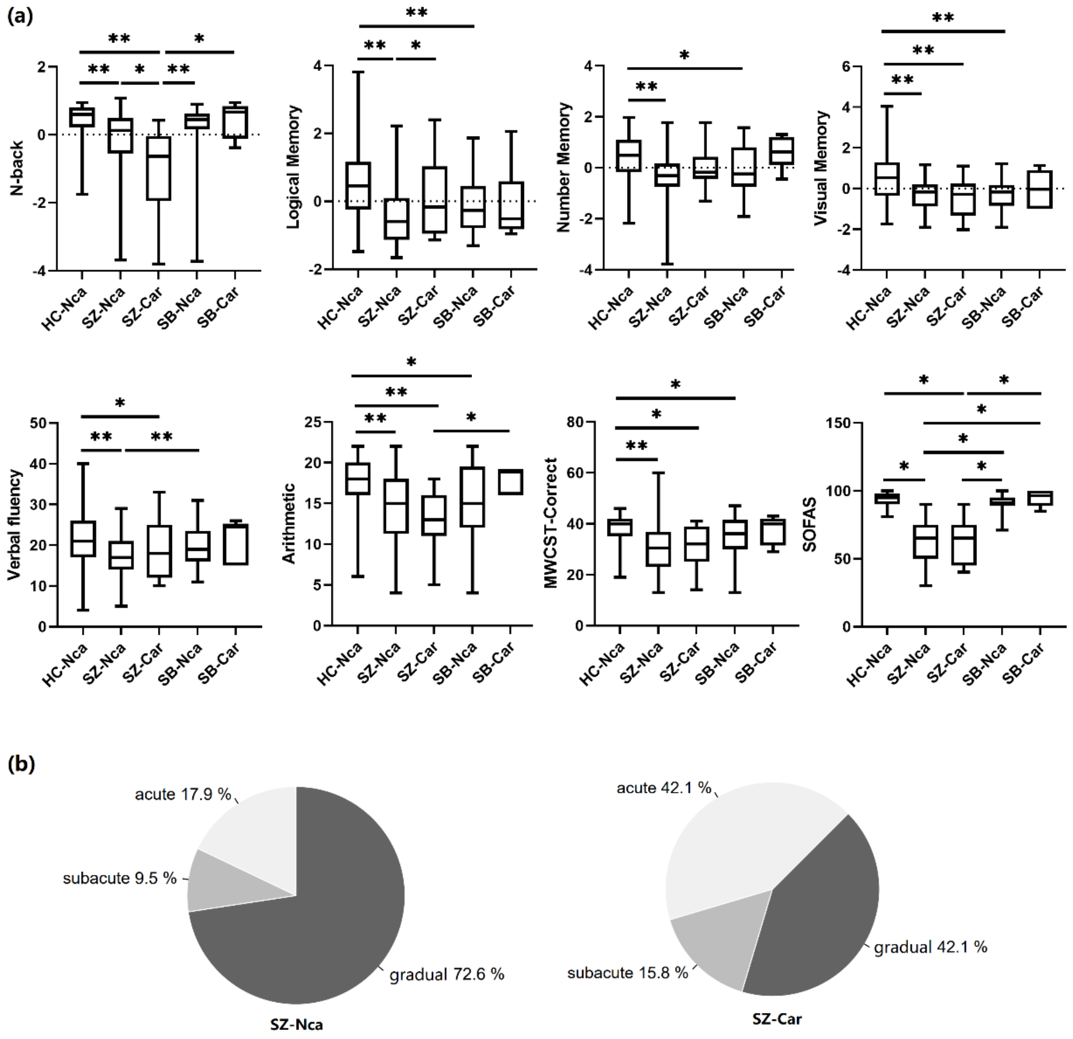

(A) Cognitive characteristics of the 5 groups. (B) Onset characteristics. Mode of SZ onset according to diagnostic thresholds: 17.9%, 9.5%, and 72.6% for acute, subacute, and gradual onset in SZ-Nca; 42.1%, 15.8%, and 42.1% in SZ-Ca. HC-Nca = healthy control group SNV20673 non-carriers; MWCST = modified Wisconsin card sorting test; SB-Nca = unaffected sibling group SNV20673 non-carriers; SB-Car = unaffected sibling group SNV20673 carriers; SOFAS = the Social and Occupational Functioning Assessment Scale; SZ-Nca = schizophrenia group SNV20673 risk allele non-carriers; SZ-Ca = schizophrenia group SNV20673 carriers.

Supplementary Material S2

Analysis in all adult samples

A total of 191 participants (60 schizophrenia patients [SZ], 99 unaffected patient siblings [SB], and 32 healthy controls [HC]) were enrolled after excluding those <18 years of age. The demographic, clinical, and cognitive characteristics of participants are shown in Table S1. There were significant sex and age differences among the groups.

Table S9 Detailed demographic, clinical, and cognitive characteristics of the participants

	**SZ (n=60)**	**HC (n=99)**	**SB (n=32)**	**F/χ2**	**P-value**
Age, years	20.5 ± 3.12	22.45 ± 3.48	24.56 ± 4.85	13.523	< 0.001
Sex	33/27	49/50	8/24	8.003	0.018
N-back (PCA Component)	-0.51 ± 1.25	0.44 ± 0.52	0.07 ± 1.14	15.395	< 0.001
Logical Memory (PCA Component)	-0.54 ± 0.74	0.56 ± 0.92	-0.08 ± 0.95	26.076	< 0.001
Digit Span (PCA Component)	-0.40 ± 0.95	0.50 ± 0.88	0.12 ± 0.95	16.067	< 0.001
Visual Memory (PCA Component)	-0.34 ± 0.77	0.61 ± 1.09	-0.30 ± 0.78	20.864	< 0.001
Arithmetic	14.31 ± 4.38	17.82 ± 3.55	15.63 ± 4.40	12.966	< 0.001
Verbal fluency	17.00 ± 6.10	22.28 ± 6.39	20.41 ± 4.93	12.594	< 0.001
MWCST	29.64 ± 7.44	38.39 ± 5.94	35.75 ± 8.64	25.213	< 0.001
SOFAS	64.08 ± 15.74	93.95 ± 4.27	93.25 ± 4.44	168.760	< 0.001
DoI, months	32.78 ± 30.16	/	/	/	/
DoM, months	22.74 ± 27.33	/	/	/	/
CPZ equivalent (×100mg/d)	2.56 ± 1.62	/	/	/	/
PANSS	67.73 ± 23.87	/	/	/	/
SSRS	12.68 ± 7.27	/	/	/	/

CPZ = chlorpromazine; DoI = duration of illness; DoM = duration of medication; HC = healthy controls; MWCST = modified Wisconsin card sorting test; PANSS = Positive And Negative Syndrome Scale; PCA = principal component analysis; SB = unaffected patient siblings; SOFAS = the Social and Occupational Functioning Assessment Scale; SSRS = the Schizophrenia Suicide Risk Scale; SZ = schizophrenia patients.

A general linear model with age and sex as covariates revealed 8 brain regions that significantly differed among the groups: bilateral middle temporal, left banks of the superior temporal sulcus, caudal middle frontal, pars opercularis, pars triangularis, supramarginal, right middle temporal, and superior temporal. In most of these 8 regions, SZ had the thinnest cortex, with SB between SZ and HC. We included these 8 brain regions in subsequent association

analyses. Due to the relatively small sample, we did not replicate the single nucleotide variant or PRS analysis.

Interestingly, following multivariate analysis with age and sex as covariates, we found that SNV20673 had a significant main effect on cortical thickness in the left pars triangularis (P_{FDR} = 0.016) (Figure S2). SNV20673 risk allele carriers had significantly thinner cortex in the right pars triangularis than non-carriers. No significant main effects were observed for other SNPs (rs35542507, rs41277477, and rs73165153).

Figure S5 Cortical thickness differences in the left pars triangularis among 5 subgroups

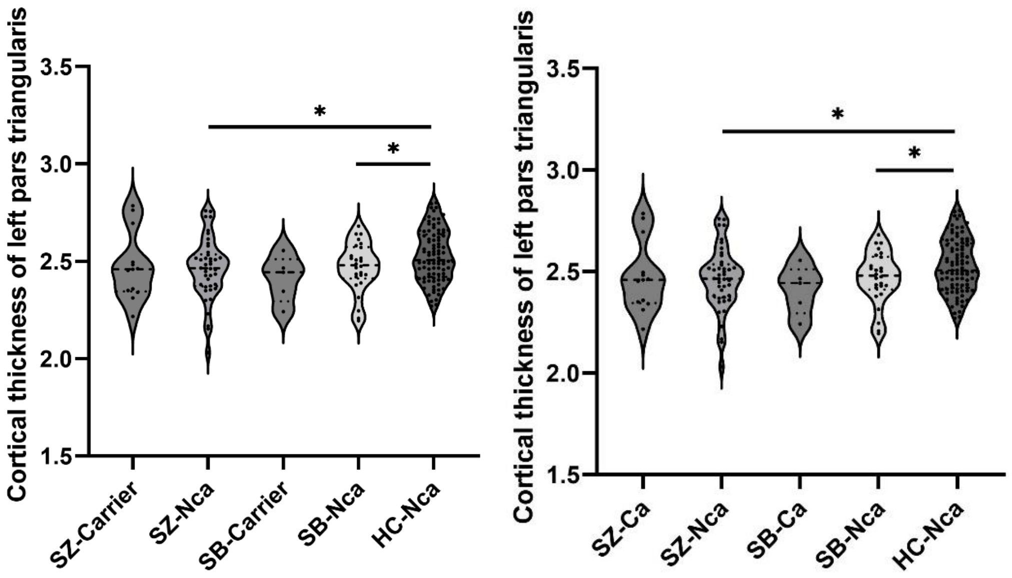

HC-Nca = healthy control group SNV20673 non-carriers; SB-Ca = unaffected sibling group SNV20673 carriers; SB-Nca = unaffected sibling group SNV20673 non-carriers; SZ-Ca = schizophrenia group SNV20673 carriers; SZ-Nca = schizophrenia group SNV20673 non-carriers. * $p<0.05$

Post-hoc analysis showed that schizophrenia group SNV20673 non-carriers (SZ-Nca) (p=0.014) and unaffected sibling group SNV20673 non-carriers (SB-Nca)(p=0.042) had significantly lower cortical thickness than healthy control group SNV20673 non-carriers (HC-Nca). No significant differences were found between SZ-Nca and SZ-Car (p=0.861), SB-Nca (p=0.986), or SB-Car (p=0.406). No significant differences were found between SZ-Car and SB-Car (p=0.394) or HC-Nca (p=0.167). No significant differences were found between HC-Nca and SB-Car (p=0.062) or between SB-Nca and SB-Car (p=0.402).

No differences were found in clinical symptom severity or mode of onset between SZ-Ca and SZ-Nca, although there were significant differences between SZ-Ca and SZ-Nca in N-back test results, with SZ-Car having lower performance. No significant differences were found between groups in the other cognitive tests. No significant correlations were found between left pars triangularis cortical thickness, cognitive tests, and clinical symptom severity in SZ.

References

1. Della Sala S, Gray C, Baddeley A, Allamano N, Wilson L. Pattern span: a tool for unwelding visuo-spatial memory. Neuropsychologia. 1999;37:1189-99.
2. Purcell S, Neale B, Todd-Brown K, Thomas L, Ferreira MA, Bender D, et al. PLINK: a tool set for whole-genome association and population-based linkage analyses. Am J Hum Genet. 2007;81:559-75.
3. Choi SW, O'Reilly PF. PRSice-2: Polygenic Risk Score software for biobank-scale data. Gigascience. 2019;8:giz082.
